# Post-traumatic stress disorder in parturients delivering by caesarean section and the implication of anaesthesia: a prospective cohort study

**DOI:** 10.1186/s12955-017-0692-y

**Published:** 2017-06-02

**Authors:** U. Lopez, M. Meyer, V. Loures, I. Iselin-Chaves, M. Epiney, C. Kern, G. Haller

**Affiliations:** 1 Department of Neurology, Neuropsychology Unit, University Hospital of Geneva, Rue Gabrielle, Perret-Gentil 4, 1211 Geneva, Switzerland; 2 Department of Anaesthesia, University Hospital of Lausanne, Rue du Bugnon 46, 1011 Lausanne, Switzerland; 3 Department of Anaesthesia Pharmacology & Intensive Care, University Hospital of Geneva, Rue Gabrielle Perret-Gentil 4, 1211 Geneva, Switzerland; 4 Department of Gynaecology & Obstetric-section, University Hospital of Geneva, Rue Gabrielle, Perret-Gentil 4, 1211 Geneva, Switzerland

**Keywords:** Post-partum post-traumatic stress disorder, Anaesthesia, Caesarean section, Risk factors

## Abstract

**Background:**

Post-traumatic stress disorder (PTSD) occurs in 1–7% of women following childbirth. While having a caesarean section (C-section) is known to be a significant risk factor for postpartum PTSD, it is currently unknown whether coexisting anaesthesia-related factors are also associated to the disorder. The aim of this study was to assess anaesthesia-linked factors in the development of acute postpartum PTSD.

**Methods:**

We performed a prospective cohort study on women having a C-section in a tertiary hospital in Switzerland. Patients were followed up six weeks postpartum. Patient and procedure characteristics, past morbidity or traumatic events, psychosocial status and stressful perinatal events were measured. Outcome was divided into two categories: full PTSD disease and PTSD profile. This was based on the number of DSM-IV criteria of the Diagnostic and Statistical Manual of Mental Disorders 4th edition (DSM-IV) present. The PTSD Checklist Scale and the Clinician Administered PTSD Scale were used for measurement.

**Results:**

Of the 280 patients included, 217 (77.5%) answered the questionnaires and 175 (62.5%) answered to an additional phone interview. Twenty (9.2%) had a PTSD profile and six (2.7%) a PTSD. When a full predictive model of risk factors for PTSD profile was built using logistic regression, maternal prepartum and intrapartum complications, anaesthetic complications and dissociative experiences during C-section were found to be the significant predictors for PTSD profile.

**Conclusion:**

This is the first study to show in parturients having a C-section that an anaesthesia complication is an independent risk factor for postpartum PTSD and PTSD profile development, in addition to known perinatal and maternal risk factors.

## Background

Childbirth is a highly emotional and challenging event in a woman’s life. However, rather than experiencing the joys of welcoming a new child, some women may experience fear for the life or wellbeing of their newborn, as well as their own. This can lead to serious psychological complications such as Post-traumatic Stress Disorder (PTSD) [1].

First identified in relation to war experiences, PTSD is an anxiety disorder that can occur after many types of traumatic events. According to the 1994 Diagnostic and Statistical Manual of Mental Disorders fourth ed. (DSM-IV) manual [2] used for the study before publication of the DSM-V manual, the disorder follows a traumatic experience usually involving threat to ones’ own life or physical integrity. It can also be associated with the witnessing of an event that involves death, injury, or threat to the physical integrity of another person (Criterion A). Response to that stressful event must include a specific number of symptoms from each of the three following criteria: (B) at least one re-experiencing symptom (i.e. the traumatic event is re-experienced as repetitive intrusive memories, flashbacks and nightmares), (C) three avoidance symptoms (i.e. avoidance of hospital or medical encountering), (D) two hyperarousal symptoms (i.e. irritability and hypervigilance). Symptoms must be present for more than 1 month (Criterion E) and must cause clinically significant distress or impairment in social, occupational or other important areas of functioning (Criterion F).

On the other hand, “Partial” PTSD, also referred to as PTSD “Profile” or “Subthreshold” PTSD, describes clinically significant PTSD symptoms in trauma-exposed persons who do not meet full criteria for PTSD [3–5]. This category deserves equal attention as full PTSD, as a number of victims suffer from severe disturbing symptoms while not fulfilling all the PTSD criteria [3, 5, 6]. For instance, significant associations have been demonstrated between PTSD profiles and increased rate of suicidal ideation [7], alcohol use [8], episodes of absence at work place or increased healthcare utilization [9]. Thus, incomplete PTSD (PTSD profile) can cause significant impairment in daily life activity [5].

In the context of childbirth, it is currently known that 1–7% of parturients will experience partial or fully characterized PTSD at any given time point following childbirth [1]. Parturients suffering from PTSD are impaired in many aspects of their daily life and have an increased risk of developing long term depression, associated with difficulties in childcare and bonding, apprehension of sexual intercourse and of future pregnancies [10–14]. Their risk of suicide is significantly increased [15]. Since the first studies by Bydlowski in 1978 [16], researchers have isolated perinatal and maternal risk factors leading to the development of PTSD [1, 10, 17–20]. These include psychiatric disorders, previous trauma, poor levels of education and social support, increased sense of loss of control and psychological distress during labour. Additional factors linked to the occurrence of neonatal complications (poor staff support as well as deliveries in emergency situations) have also been identified [1, 12, 19, 20]. Amongst these, childbirth following a scheduled or emergency C-section appears as one of the top predictors for PTSD occurrence [19, 21]. However, it is unknown whether a C-section per se or if concomitant factors linked to it, particularly anaesthesia, play a role in the occurrence of a PTSD.

The aim of this study was to measure in a prospective cohort of parturients having a delivery by C-section, known risk factors for postpartum PTSD and assess the specific impact of anaesthesia-linked factors on the disease’s development. As major suffering and psychological sequelae can emanate from partial PTSD [3, 5, 6], we measured the full acute PTSD disease, as well as the acute partial PTSD symptoms according to Olde et al. [1] as “PTSD profile”, which include the DSM criteria B, C and D only.

Parturients with PTSD profile were included in the study as well as those who had the full spectrum of DSM-IV criteria for PTSD in order to include the maximum of patients with severe psychological morbidity from the traumatic experience. Moreover, the group with PTSD profile was selected in order to emphasize the continuum of this disorder and the need to prevent and manage partial symptoms of PTSD as well.

## Methods

This study was performed between 2008 and 2011 at the maternity of the University Hospital of Geneva, a tertiary referral hospital in Switzerland. It was approved by the Geneva University Hospital Ethics Committee (Switzerland). Approval N°-CER 07–240- Ref Matped 07–045 (7/07/2008)- Chairperson Prof Michel Boulvain.

### Participants

We included in a prospective cohort all parturients undergoing an emergency or scheduled C-section under general or neuraxial anaesthesia during weekdays and who provided written consent for the study. In particular situations (i.e. extreme emergency surgery) when consent could not be provided prior the C-section, all of the demographic and procedure-related data was temporarily recorded, and approval requested afterwards. If patients did not consent to the study, the data was systematically deleted. We excluded all patients who were unable, because of a language barrier or serious cognitive impairment, to answer to the different questionnaires used for PTSD measurement. We also excluded patients transferred during weekends or in emergency situation from other areas of Switzerland, or neighbouring countries (France, Italy) and unlikely to be included or followed up.

### Variables collected

Variables measured included *demographic characteristics and psychosocial factors* (such as age, origin and marital status, and education level) as well as *anaesthesia-related procedure characteristics* (type of anaesthesia, degree of certification of anaesthetist, use of adjuvant anaesthesia, quality of anaesthesia information provided) which were extracted from patients’ charts. The women were usually given information before the C-section on neuraxial or general anaesthesia with its risks and side effects, unless the degree of emergency rendered it impossible.

**Fig. 1:**
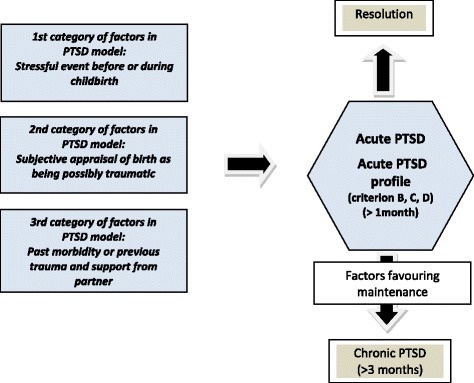
Model of elements leading to PTSD development

The other variables collected were classified according to the model developed by L.B. Andersen et al. [19] for PTSD following childbirth. This model (Fig. 1) identifies three categories of factors that can lead to a PTSD or PTSD profile in parturients:

1) *A highly stressful event before or during childbirth* (i.e. emergency C-section, intra/postpartum complication);

2) *A subjective appraisal of birth as being possibly traumatic* (i.e. negative emotions and perceptions during labor such as feeling of loss of control or peritraumatic dissociation);

3) *A group of various related factors such as past morbidity* (psychiatric/other) *or previous trauma and support from partner*.

In our study, the first factor included the following variables: Unexpected prematurity; Emergency caesarean section, Long duration of surgery (>50 min), Maternal prepartum complications, Intra/postpartum complications, Anaesthetic complication, and Neonatal complication. The second factor included dissociative experiences during C-section and the last one included the following variables: Past psychiatric disorder, Past traumatic experience, Any other somatic disorder, Past pregnancy abortion, Past caesarean section, and Support from partner during pregnancy and C-section.

Variables related to each of these three categories of factors were prospectively collected by the research staff or the anaesthetist in charge, during or within 48 h following the procedure. Missing information was extracted retrospectively from handwritten or electronic patient records and incident reports by research staff.

### Description of usual care

The anaesthetist in charge of the patient chose the type of anaesthesia. Spinal anaesthesia was proposed routinely for elective procedures unless contraindicated. For emergency procedures anaesthesia was decided according to the degree of emergency: spinal or pre-existing peridural if the delivery could wait more than 30 min and general anaesthesia if delivery was urgent or according the maternal health status. The anaesthesia staff was composed of minimum one anaesthesiologist (certified or in training) and a certified anaesthetic nurse.

Information and frequent updates were given during the procedure under spinal anaesthesia on current medical situation, normal sensations, causes and treatments given concerning hypotension, nausea, vomiting, itching, shaking, pain, allergic reactions or unusual bleeding. Updates on the newborn’s wellbeing were given frequently. Postpartum feedback and explanation of anaesthesia-linked complication sand its treatments during and after C-section were routinely given 24-48 h postpartum and discussed.

### Measurement tools

#### Demographic characteristics and psychosocial factors

For the level of education we used the International Standard Classification of Occupations- ISCO-88 [22] and for the country of origin we used continents. To measure socio-economical level, we used a surrogate, the type of insurance premium paid by patients. In Switzerland, these premiums are very high if patients are paying for private or semi-private care.

#### Anaesthesia-related procedure characteristics

Type of anaesthesia was divided into epidural or combined spinal-epidural (Tuohy 18 gauge/Whitacre 27/25 gauge needle through needle), spinal anaesthesia (Whitacre 25/27 gauge) and general anaesthesia. If a neuraxial anaesthesia had to be converted into a general anaesthesia it was considered as a general anaesthesia procedure. The quality of anaesthesia information provided to parturients was assessed using an institution-based satisfaction survey. Parturients were also asked whether they would have liked additional sedation during the procedure as the use of sedative drugs during C-Section is not part of standard care.

#### First category of factors in PTSD model: Stressful event before or during childbirth

Maternal prepartum complications were all complications occurring during pregnancy (i.e. pre-eclampsia, diabetes) and intra or postpartum complications, all those occurring during or immediately after the C-section (i.e. haemorrhage, sepsis).

Anaesthesia complications were defined as any undesirable events related to anaesthesia and occurring during or shortly after procedure. These included the following events: 1) Severe nausea and vomiting (repeated episodes of nausea and/or expulsion of gastric contents, at least 6 h apart within 24 h and requiring treatment with at least three doses of at least two different classes of antiemetic medication); 2) Failed spinal/epidural anaesthesia leading to intraoperative pain and requiring rescue medications or conversion from spinal/epidural; 3) Unintentional dural puncture followed by severe postpartum postdural puncture headache; 4) Traumatic needle insertion with pain during procedure and/or residual cutaneous haematoma; 5) Neurological injuries (central or lower extremity motor and sensory dysfunction linked to spinal/epidural anaesthesia).

Any newborn requiring unexpectedly advanced support and admission into the neonatal care unit was considered as having a neonatal complication.

#### Second category of factors in PTSD model: Subjective appraisal of birth as being possibly traumatic

We measured peritraumatic dissociation experienced during C-section using the ten question of the Peritraumatic Dissociative Experience Questionnaire (PDEQ) [23]. Peritraumatic dissociation is conceptualized as a distorted way of processing information during or shortly after trauma exposure. Trauma victims often report alterations in perception of time, place and persons. These alterations include the perception of time slowing or rapidly accelerating, out-of-body experiences, detachment from the on-going experience and profound unreality about the occurrence of the event [23].

#### PTSD and support from partner assessment

For PTSD and PTSD profile screening, we used a mail-based questionnaire, the Patient PTSD Checklist Scale (PCLS) [24] which is an internationally recognized screen tool for PTSD, combined to a short ten-minute standardized clinical interview based on all the DSM-IV PTSD criteria. This was performed within 6 weeks by phone by a trained psychologist. This standardized interview also included generic questions asking whether parturients felt supported or not from their partner during the pregnancy or caesarean section. For those who provided a positive PCLS score (≥44) [25] or had clinical criteria suggesting PTSD following the 10-min short interview, a semi-structured one-hour phone interview using the Clinician Administered PTSD Scale (CAPS) [26] was performed by a second psychologist. The CAPS scale is considered as the “gold standard” and most rigorous tool for PTSD diagnosis [27] as it takes into account all the DSM-IV criteria for PTSD. Thus, in this study, as recommended in the literature [1], a combination of standardized clinical interviews and standardized questionnaire was used for the assessment of PTSD. Details of questionnaires’ type and timing of use are described in Table 1.

**Table 1:** Study population characteristics and timing of assessment

Variable	N (%) (*N* = 175)
Age	
< 31 yr	49 (28.0)
≥ 31 yrs. and <35 yrs	61 (34.8)
> 36 yrs	65 (37.2)
Pregnancy	
Number of gestations	
1	63 (36.0)
2	52 (29.7)
≥ 3	60 (34.3)
Number of childbirths	
0	87 (49.7)
1	63 (36.0)
≥ 2	25 (14.3)
Weeks of gestation at delivery	
< 31 yr	53 (30.2)
≥ 31 yrs. and <35 yrs	44 (25.1)
> 36 yr	76 (44.1)
Missings	2 (0.6)
Timing of Questionnaire Administration	
Anaesthesia information; Peritraumatic dissociation (PDEQ) ≤ 48 h	175 (100)
PCLS; Standardized clinical interview based on the DSM-IV PTSD criteria ≤6 weeks	175 (100)
CAPS (only when the PCLS or the clinical evaluation revealed the presence of PTSD symptoms) ≤ 6 weeks	171 (97.7)^a^

^a^4 (1.4%) parturients refused or were unable to answer to the one-hour phone interview

Depending on the characteristics of symptoms reported, patients were considered as having either an acute PTSD profile (criterion B, C, D) or a full acute PTSD disease. This was done during a consensus meeting that included a third psychologist who had not participated in the telephone interviews but who crosschecked all study outcomes. All questionnaires and data collected or extracted from patient files were recorded on paper-based standardised forms.

These were then transformed into an electronic format by professional data coders (DataConversion®) and validated for internal consistency before being integrated on a basic Excel 2010 (Microsoft Corp., Redmond, WA, USA) sheet.

### Statistical analysis

Data were then cleaned and corrected before analysis for double entries, illogical and missing values before being transformed and recorded on a statistical computer program SPSS 17.0 (SPSS Inc., Chicago, USA) and STATA (version 10.1, Stata Corp LP College Station-Texas, US). For descriptive analysis we used frequencies and percentages with 95% confidence interval. Continuous variables such as age, number of gestations, parity, duration of surgery and the PDEQ questionnaire score results were transformed into categorical variables according to statistical distribution. With a prevalence of 2.7%, an alpha error of 0.05 and a power of 0.80, a study sample of 170 patients was necessary to detect a minimal difference of 15% between groups.

We first performed a univariate analysis comparing all patients’ demographic, socio-economic characteristics, co-morbidities, treatments, complications, dissociative experiences during C-section and other possible predictors of postpartum psychological complications [19] with our study outcomes, PTSD and PTSD profile. Chi-square, Fisher’s exact test, or binary logistic regression was used, and the derived odds ratio (OR) with 95% confidence interval (CI) calculated to assess differences between groups. To identify independent risk factors more specifically related to anaesthesia and the type of care provided to parturients, we used multivariable analyses with logistic regression. We built multivariable models using a forward selection technique and incorporated into the model all univariate risk factors with a *P* value <0.10 or those with a strong clinical significance according to Andersen’s model for PTSD (i.e. obstetrics or anaesthesia related complications, marital status).

To minimise the risk of overfitting, we limited the number of variables included in the model by combining variables together (i.e. pre and intra-partum obstetric complications) and choosing only the most significant and/or representative variables of the Andersen’s model. We have also tested the level of optimism of the predictive performance *(C-index)* of the final regression model using bootstrapping techniques [28]. The significance of the Hosmer-Lemeshow goodness-of-fit test was 0.30 and the C-index of the PTSD profile model was 0.795. When corrected for optimism, it was found to be at 0.730. Final results are expressed as adjusted 95% CI and *P* values. A *P*-value of <0.05 was considered statistically significant.

## Results

**Fig. 2:**
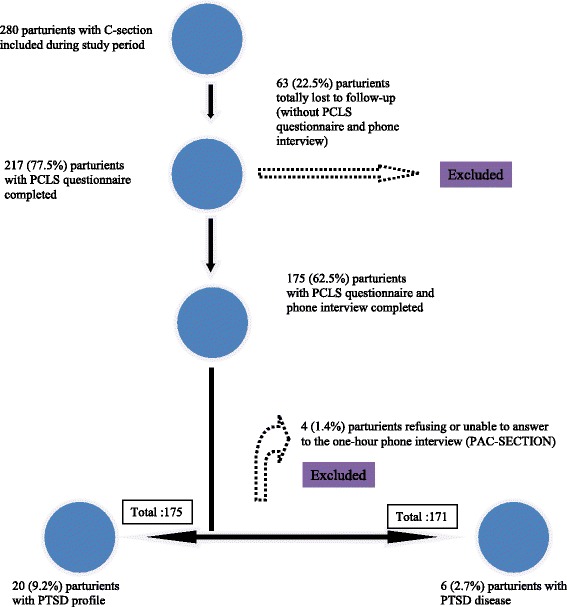
Study flow-chart and incidence of PTSD and PTSD profile

We enrolled in our study cohort, 280 women having a C-section and willing to participate in the study. Of these, 217 (77.5%) answered to the PCLS questionnaire and 175 (62.5%) had an additional phone standardized clinical interview performed. We identified 6 parturients (2.7%) with a confirmed PTSD and 20 (9.2%) with a PTSD profile (Fig. 2). Study population characteristics are detailed in Table 1. Most parturients (62.8%) were younger than 35 years and primipara or secondipare (65.7%). Risk factors for acute PTSD identified following univariate analysis (Table 2) were a non-European status, having a past significant somatic disease and a high level of dissociative reactions during C-section. Being married compared to single decreased the risk of PTSD and there was a higher number of patients with a PTSD who wished receiving additional sedation during the procedure.

**Table 2:** Univariate analysis for risk factors of acute PTSD disease

Risk factors	No PTSD *n* = 165 N (%)	Confirmed PTSD *n* = 6 N (%)	OR (95% CI)	*P* ^a^
Demographic characteristics and psycho-social factors				
Age				┐
< 31 yr	44 (26.7)	2 (33.3)	1 (reference)	0.74†
≥ 31 yrs. and <35 yrs	59 (35.8)	2 (33.3)	0.74 (0.10–5.50)	┘
> 36 yrs	62 (37.6)	2 (33.3)	0.71 (0.09–5.23)	
Pregnancy				
Past pregnancy	105 (63.6)	5 (83.3)	2.85 (0.32–25.0)	0.32
Past childbirth	86 (52.1)	3 (50.0)	0.91 (0.18–4.68)	0.91
Origin				
European	138 (83.6)	3 (50.0)	5.1 (1.0–26.6)	**0.03**
Non-European	27 (16.4)	3 (50.0)		
Marital Status				
Married/In couple	158 (96.3)	2 (33.3)	0.07 (0.012–0.49)	**<0.001**
Single	6 (3.7)	4 (66.7)		
Education level parturient (ISCO-88)				
None to second level	73 (49.7)	4 (66.7)	0.49 (0.08–2.77)	0.41
Third and fourth level	74 (50.3)	2 (33.3)		
Education level partner (ISCO-88)				
None to second level	71 (55.0)	2 (50.0)	1.22 (0.16–8.95)	0.84
Third and fourth level	58 (45.0)	2 (50.0)		
Insurance				
None or standard	145 (87.9)	5 (83.3)	1.45 (0.16–13.05)	0.73
High Premium	20 (12.1)	1 (16.7)		
Anaesthesia-related procedure characteristics				
Type of anaesthesia				
General	6 (3.6)	0	-	┐
Spinal	100 (60.6)	5 (83.3)	1.0 (reference)	0.52†
Spinal-epidural	59 (35.8)	1 (16.7)	0.33 (0.03–2.97)	┘
Certified anaesthetist	87 (52.7)	3 (50.0)	0.89 (0.17–4.57)	0.89
Adjuvant to anaesthesia				
Midazolam, clonidine, ketamine	18 (10.9)	1 (16.7)	1.63 (0.18–14.77)	0.65
Would have liked additional sedation	20 (12.3)	3 (50.0)	7.10 (1.34–37.61)	**0.008**
Poor anaesthesia information(before, during, after procedure)	21 (13.0)	1 (16.7)	1.34 (0.14–12.06)	0.79
1st category of factors in PTSD model: Stressful event before or during childbirth				
Unexpected prematurity	24 (14.7)	2 (33.0)	2.8 (0.5–16.6)	0.21
Emergency caesarean section	88 (53.3)	2 (33.3)	0.43 (0.07–2.45)	0.43
Long duration of surgery (>50 min)	66 (40.2)	1 (20.0)	0.37 (0.04–3.39)	0.36
Maternal prepartum complications	97 (58.8)	5 (83,3)	3.50 (0.40–30.67)	0.22
Intra/postpartum complications	103 (62.4)	5 (83.3)	3.01 (0.34–26.36)	0.29
Anaesthetic complication	17 (10.3)	2 (33.3)	4.35 (0.74–25.55)	0.07
Neonatal complication	26 (16.8)	5 (25.0)	1.65 (0.55–4.95)	0.36
2nd category of factors in PTSD model: Subjective appraisal of birth as being possibly traumatic				
Dissociation during C-section (PDEQ score)				
Low /Intermediate degree of dissociation	172 (72.0)	1 (16.1)	12.8 (1.46–13.38)	0.008
High degree of dissociation	45 (28.0)	5 (83.3)		
3rd category of factors in PTSD model:Past morbidity or previous trauma and support from partner				
Past morbidity or previous trauma				
Past psychiatric disorder	18 (11.0)	1 (16.7)	0.3 (0.05–3.1)	0.35
Past traumatic experience	8 (7.6)	1 (11.1)	3.1 (0.3–31.6)	0.30
Any other somatic disorder	104 (63.4)	6 (5.5)	1.0 (1.01–1.10)	0.05
Past pregnancy abortion	76 (46.1)	2 (33.3)	0.58 (0.10–3.28)	0.53
Past caesarean section	56 (33.9)	3 (50.0)	1.94 (0.38–9.95)	0.41
Support from partner				
During pregnancy	158 (96.9)	6 (100)	1.0	0.63
During caesarean section	147 (89.6)	6 (100)	1.0	0.43

^a^ *Fisher exact test for values < 5*

†*P* value for χ2 test for linear trend

*P* value <0.05 in bold

┐┘*P* value of Chi square tests for trend

Risk factors for acute PTSD profile (Table 3) were the presence of a stressful factor such as prepartum, intrapartum or postpartum maternal complications, a high level of dissociative reactions during C-section and having a past significant somatic disease.

**Table 3:** Univariate analysis for risk factors of acute PTSD profile

Risk factors	No PTSD-profile *n* = 155 N (%)	Confirmed PTSD-profile *n* = 20 N (%)	OR (95% CI)	*P value* ^a^
Demographic characteristics and psycho-social factors				
Age				┐
< 31 yr	43 (27.7)	6 (30.0)	1 (reference)	0.88†
≥ 31 yrs. and <35 yrs	55 (35.5)	6 (30.0)	0.78 (0.23–2.59)	┘
> 36 yrs	57 (36.8)	8 (40.0)	1.0 (0.32–3.11)	
Pregnancy				
Past pregnancy	100 (64.5)	12 (60.0)	0.82 (0.31–2.14)	0.32
Past childbirth	81 (52.3)	7 (35.0)	0.14 (0.18–1.29)	0.91
Origin				
European	128 (82.6)	17 (85.0)	0.83 (0.22–3.05)	0.78
Non-European	27 (17.4)	3 (15.0)		
Marital Status				
Married/In couple	148 (96.1)	18 (90.0)	0.36 (0.06–1.94)	0.22
Single	6 (3.9)	2 (10.0)		
Education level parturient (ISCO-88)				
None to second level	68 (48.9)	12 (66.7)	0.47 (0.17–1.34)	0.15
Third and fourth level	71 (51.1)	6 (33.3)		
Education level partner (ISCO-88)				
None to second level	66 (54.1)	9 (60.0)	0.78 (0.26–2.34)	0.66
Third and fourth level	56 (45.9)	6 (40.0)		
Insurance				
None or standard	135 (87.1)	18 (90.0)	0.75 (0.16–3.47)	0.71
High Premium	20 (12.9)	2 (10.0)		
Anaesthesia-related procedurecharacteristics				
Type of anaesthesia				
General	5 (3.2)	2 (10.0)	1 (reference)	┐
Spinal	96 (61.9)	10 (50.0)	0.26 (0.04–1.52)	0.27†
Spinal-epidural	54 (34.8)	8 (40.0)	0.37 (0.06–2.24)	┘
Certified anaesthetist	81 (52.3)	11 (55.0)	1.11 (0.43–2.84)	0.81
Adjuvant to anaesthesia				
Midazolam, clonidine, ketamine	17 (11.0)	2 (10.0)	0.90 (0.19–4.23)	0.89
Would have liked additional sedation	18 (11.8)	5 (25.0)	2.48 (0.80–7.64)	0.10
Poor anaesthesia information(before, during, after procedure)	18 (11.8)	5 (25.0)	2.48 (0.80–7.64)	0.10
1st category of factors in PTSD model:Stressful event before or during childbirth				
Unexpected prematurity	22 (14.3)	4 (21.1)	1.6 (0.48–5.26)	0.43
Emergency caesarean section	80 (51.6)	13 (65.0)	1.74 (0.65–4.59)	0.25
Long duration of surgery (>50 min)	64 (41.3)	7 (38.9)	0.90 (0.33–2.46)	0.84
Maternal prepartum complications	88 (56.8)	17 (85,0)	4.31 (1.21–15.33)	**0.015**
Intra/postpartum maternal complications	95 (61.3)	17 (85.0)	3.57 (1.0–12.73)	**0.03**
Anaesthetic complication	15 (9.7)	4 (20.0)	2.33 (0.69–7.88)	0.16
Neonatal complications	26 (1.8)	5 (25.0)	1.65 (0.55–4.95)	0.36
2nd category of factors in PTSD model: Subjective appraisal of birth as being possibly traumatic				
Dissociation during C-section (PDEQ score)				
Low degree of dissociation	57 (37.5)	2 (10.5)	1 (reference)	┐
Intermediate degree of dissociation	51 (33.6)	8 (42.1)	4.47 (0.90–22.03)	**0.02**†
High degree of dissociation	44 (28.9)	9 (47.4)	5.83 (1.19–28.35)	┘
3rd category of factors in PTSD model: Past morbidity or previous trauma and support from partner				
Past morbidity or previous trauma				
Past Psychiatric disorder	18 (11.7)	1 (5.0)	0.39 (0.05–3.1)	0.36
Past traumatic experience	8 (7.6)	1 (9.1)	1.2 (0.13–10.7)	0.86
Any other somatic disorder	94 (61.0)	17 (85.0)	3.6 (1.01–12.87)	**0.03**
Past pregnancy abortion	73 (47.1)	7 (35.0)	0.60 (0.22–1.52)	0.53
Past caesarean section	53 (34.2)	6 (30.0)	0.82 (0.30–2.27)	0.70
Support from partner				
During pregnancy	149 (97.3)	19 (95.0)	0.51 (0.05–4.80)	0.95
During caesarean section	138 (89.6)	18 (90.0)	1.04 (0.22–4.91)	0.54

^a^ *Fisher exact test for values < 5*

†*P* value for χ2 test for linear trend

*P* value <0.05 in bold

┐┘*P* value of Chi square tests for trend

When a full multivariable model for risk factors for acute PTSD profile was built (Table 4), maternal prepartum, intrapartum or postpartum complications, anaesthetic complications and dissociative experiences during C-section were found to be the most significant predictors. These were independent of the quality of the information provided by the anaesthesia team or parturients’ family status.

**Table 4:** Multivariable analysis for risk factors of acute PTSD profile

Risk factors	OR (95% CI)	*P*
Demographic characteristics and psycho-social factors		
Married/In couple vs Single	0.61 (0.09–4.14)	0.62
Anaesthesia-related procedure characteristics		
Poor anaesthesia information (before, during, after procedure)	2.86 (0.71–11.49)	0.13
First category of factors in PTSD model: Stressful event before or during childbirth		
Maternal prepartum intra/postpartum complications	4.66 (1.54–14.06)	**<0.010**
Anaesthetic complications	4.32 (1.04–17.87)	**0.04**
Second category of factors in PTSD model: Subjective appraisal of birth as being possibly traumatic		
High /intermediate or low degree of dissociation during C-section (PDEQ score)	2.14 (1.08–4.25)	**0.02**

*P* value <0.05 in bold

We also found that 63 (22.5%) patients were totally lost to follow up. These parturients did not complete the PCLS questionnaire and had no phone interview (Fig. 2). 49 patients (17.5%) completed the PCLS questionnaire but had no phone interview. When assessing the characteristics of parturients without phone interview (109 patients), we found that they were more often single, had lower education level and had more often past traumatic experiences and abortion (Table 5).

**Table 5:** Comparison of patients with or without follow up for PTSD disease assessment

Risk factors	Patients with follow up for PTSD disease assessment (with PCLS and phone interview) *n* = 171 N (%)	Patients without follow up for PTSD disease interview (without phone interview) *n* = 109 N (%)	*P value* ^a^
Demographic characteristics and psycho-social factors			
Age			┐
< 31 yr	46 (26.9)	41 (37.6)	0.09†
≥ 31 yrs. and <35 yrs	61 (35.7)	39 (35.8)	┘
> 36 yrs	64 (37.4)	29 (26.6)	
Pregnancy			
Past pregnancy	110 (64.3)	82 (75.2)	0.05
Past childbirth	89 (52.0)	60 (55.0)	0.91
Origin			
European	141 (82.5)	79 (73.1)	0.06
Non-European	30 (17.5)	29 (26.9)	
Marital Status			
Married/In couple	160 (93.5)	96 (88.1)	**0.02** ^a^
Single	8 (6.5)	13 (11.9)	
Education level parturient (ICSO 88)			
None to second level	77 (50.3)	60 (63.8)	**0.03** ^a^
Third and fourth level	76 (49.7)	34 (36.2)	
Education level partner (ICSO 88)			
None to second level	73 (54.9)	42 (51.2)	0.60
Third and fourth level	60 (45.1)	40 (48.8)	
Insurance			
None or standard	150 (87.7)	101 (92.7)	0.18
High Premium	21 (12.3)	8 (7.3)	
Anaesthesia-related procedure characteristics			
Type of anaesthesia			
General	6 (3.5)	6 (5.5)	┐
Spinal	105 (61.4)	61 (56.0)	0.55†
Spinal-epidural	60 (35.1)	42 (38.5)	┘
Certified anaesthetist	90 (52.6)	60 (55.0)	0.69
Adjuvant to anaesthesia			
Midazolam, clonidine, ketamine	19 (11.1)	6 (5.5)	0.10
Would have liked additional sedation	23 (13.7)	13 (12.1)	0.71
Poor anaesthesia information(before, during, after procedure)	22 (13.1)	20 (18.5)	0.22
1st category of factors in PTSD model: Stressful event before or during childbirth			
Unexpected prematurity	26 (15.4)	10 (9.3)	0.14
Emergency caesarean section	90 (52.6)	66 (60.6)	0.19
Long duration of surgery (>50 min)	67 (39.6)	47 (43.9)	0.48
Maternal prepartum complications	102 (59.6)	60 (55.0)	0.44
Intra/postpartum complications	108 (63.2)	74 (67.9)	0.41
Anaesthetic complication	19 (11.1)	9 (8.3)	0.43
Neonatal complication	31 (18.1)	20 (18.3)	0.96
3rd category of factors in PTSD model: Past morbidity or previous trauma and support from partner			
Past morbidity or previous trauma			
Past psychiatric disorder	19 (11.2)	9 (8.5)	0.47
Past traumatic experience	9 (7.9)	14 (18.4)	**0.02** ^a^
Any other somatic disorder	110 (64.7)	73 (68.2)	0.35
Past pregnancy abortion	78 (45.6)	65 (59.6)	**0.02** ^a^
Past caesarean section	59 (34.5)	39 (35.8)	0.82
Support from partner			
During pregnancy	164 (97.0)	106 (99.1)	0.26
During caesarean section	153 (90.0)	89 (84.0)	0.13

^a^ *Fisher exact test for values < 5*

†*P* value for χ2 test for linear trend

*P* value <0.05 in bold

┐┘*P* value of Chi square tests for trend

## Discussion

In the current study, we found an incidence of 2.7% of full acute PTSD and 9.2% of acute PTSD profile, in accordance with previous studies [1]. Significant risk factors for full acute PTSD or acute PTSD profile included: *demographic characteristics* (such as non-European status and being single), *stressful event before or during childbirth* (such as obstetrical and anaesthetic complications), *subjective appraisal of birth as being possibly traumatic* (such as a high level of dissociative reactions during C-section) and *past morbidity* (such as having a past significant somatic disease). More specifically, *stressful factors related to childbirth* and *subjective appraisal of birth as being possibly traumatic* were the most significant predictors of acute PTSD profile. These results are in line with the model developed by L.B. Andersen et al. [19] following a large meta-analysis of studies on PTSD in obstetrics. They confirm that PTSD is the result of a combination of several risk factors that can be aggregated into three main categories: 1) traumatizing events before or during childbirth, 2) subjective perception of these events as being highly traumatic, 3) predisposing psychological factors such as previous psychological trauma or past psychiatric disorder. Among these elements, the first and the second ones are, as demonstrated in our study, the most important category of factors for PTSD development.

In our study, we identified obstetrical and anaesthetic complications as being predictive factors of PTSD profile. This can be explained by the fact that these complications are potentially dangerous for the mother and/or for the baby, and are therefore and unsurprisingly associated with a PTSD profile. However, two important risk factors identified by previous studies, emergency situation and neonatal complications [19, 20, 29], did not prove significant in predicting acute PTSD profile or acute PTSD disease in our study. This could be explained by a possible lack of study power due to the limited number of disorders identified in our cohort. Nevertheless our study is the first to our knowledge, to show that anaesthesia plays a non-negligible part in it. Interestingly, the type of anaesthesia, whether general or neuraxial, had no significant impact on further acute PTSD profile or acute PTSD disease. In addition, the management of anaesthesia by an anaesthetist in training and poor information during the procedure given by the anaesthesia staff didn’t prove to increase the risk for PTSD. However, if any complication due to anaesthesia occurred (i.e. pain during surgery, nerve injury) the risk for a PTSD profile occurrence was higher.

Previous literature [18, 20] has suggested that a C-section was one of the top predictors for postpartum PTSD and depression. Our study, by analysing exclusively patients having a C-section for childbirth, shows that this paradigm should be probably refined. A number of factors are associated to the decision and completion of a C-section. These include neonatal malposition or distress, past or current maternal complications and patient willingness to avoid the subjective trauma of childbirth. Furthermore, a C-section is always associated with anaesthesia. Therefore, rather than the C-section itself, it is quite likely that all of the factors leading to, or around the C-section, contribute to the occurrence of PTSD or PTSD profile.

It is well described in the anaesthesia literature that intra-operative awareness can lead to PTSD in a number of patients [30–32]. Our study shows however that other complications related to anaesthesia can also lead to PTSD disease or profile. A failed spinal anaesthesia with intraoperative pain or a severe postpartum postdural puncture headache can also increase the risk of developing PTSD disease or profile. It is likely that these complications contribute to the emergence of negative emotions such as pain and distress, all known to be associated with PTSD [19, 33]. This shows that the presence of a potentially traumatic event including anaesthesia complication is a key factor for the development of a PTSD.

In the model developed by L.B. Andersen et al. [19], *subjective appraisal of birth as being traumatic* which include subjective distress during labour, such as loss of control, fear for oneself and/or the baby and perinatal dissociation, is another important factor predicting PTSD following labour and delivery. In the literature, it is increasingly recognized that these subjective factors play an important role in the development of a PTSD [34, 35]. The evidence for the importance of the individual appraisal of the event is highlighted in our study by the highly significant association found between the *negative appraisals of the trauma*, i.e. dissociative reactions by patients during the C-section, and the presence of a full and partial acute PTSD. More specifically, this risk factor proved to be a significant predictor of PTSD profile both in the univariate and multivariable analysis, as well as PTSD in the univariate model. This result is in accordance with the model developed by L.B. Andersen et al. [19] which shows that subjective distress during childbirth is one of the most important factor of the development of PTSD. It may suggest that these patients have insufficient coping mechanisms available [36]. The request for further sedation by these patients can be understood as an overwhelmed coping capacity resulting in a strong feeling of dissociation. However, when sedation was administered during the procedure, our study results show that this did not help preventing PTSD. Therefore, further sedation of the mother after delivery, whether asked by the patient or proposed by the staff, may help diminishing current anxiety symptoms but not preventing psychological consequences of poorly managed traumatic experiences.

A number of weaknesses of this study should be mentioned. First, due to strict inclusion and voluntary participation criteria, the number of patients followed up in our cohort was limited. Although, our incidence of 2.7% of PTSD is similar to the one described in previous studies [1], it is likely that a number of acknowledged risk factors predicting PTSD profile or PTSD such as emergency situation or neonatal complications did not prove significant because of a lack of study power. This could explain why some risk factors identified by previous studies [19, 20, 29] such as emergency situation and neonatal complications were not significant in our study. Secondly, as a number of patients from foreign origins or admitted during weekends were not included in the study, a selection bias cannot be excluded and may also explain why “emergency” did not appear as a significant risk factor for PTSD. Thirdly, a number of patients (22.5%) were totally lost to follow-up. Although a 77.5% retention rate is considered as acceptable for cohort studies [37], the fact that an additional proportion of the patients (17.5%) could not be interviewed for PTSD disease assessment, reduced study power for this outcome. When comparing patient characteristics between those who completed the study and those who could not be interviewed for PTSD disease assessment, it appeared that patients lost to follow up were more often single, had lower education level and had more often past traumatic experiences and abortion. The later are known risk factors for PTSD [19]. Thus it cannot be excluded these patients had subclinical depression or PTSD symptoms such as avoidance and did not want to be further interviewed. This is likely to have biased study results towards less severe cases of PTSD and artificially decreased its true incidence. Fourth, as telephone interviews were used to diagnose PTSD, a measurement bias cannot be excluded. To minimise this risk, we used a validated diagnostic method based on a combination of clinical interviews and standardised questionnaires [1], and all cases were discussed with a second psychologist to confirm diagnosis. Fifth, due to the small sample size of the full PTSD group, no comparison could be done with the partial PTSD group. Thus, the degree to which parturients with partial PTSD are comparable to those with a full PTSD remains unknown, as well as the specific mechanisms underlying these two categories of PTSD. These two groups were included in the present study as recommended in the literature [5]. Indeed, partial PTSD, as well as full PTSD, can be associated with significant impairment and can be chronic [5], which emphasize the importance of including this group in the present study. Further research should be done in order to better understand the characteristics of the whole spectrum of PTSD following childbirth, its repercussions and the need to treat it. Finally, in the present study, only acute PTSD and acute PTSD profile were measured. Thus, no conclusion can be drawn about the potential risk of parturients developing chronic PTSD. To address this issue, longitudinal studies are needed in order to characterize the course of full and partial PTSD and their associated impairment.

Despite these weaknesses, we identified a number of risk factors for acute PTSD and acute PTSD profile in line with existing literature and managed to show that anaesthesia complications are associated with postpartum PTSD. Future studies should look at the development of a predictive score for parturients at high risk of developing a PTSD in order to identify and treat this complication preferably in the immediate postpartum period.

## Conclusion

This study identifies predictors for acute postpartum PTSD profile and acute PTSD after delivery by C-section that can be easily recognised before or during the surgical procedure, particularly as spinal anaesthesia is most often used. In patients with a high degree of dissociative experiences during the C-section, or when maternal or anaesthesia complications occur, the risk for a postpartum PTSD profile increases significantly. These patients are likely to benefit from a close follow up by members of the obstetrics and/or anaesthesia team. If needed, intensive psychological support should be put in place as soon as possible.

## Abbreviations

CAPS: Clinical administered PTSD scale
CI: Confidence interval
C-section: Caesarean section
DSM-IV: Diagnostic and statistical manual of mental disorder 4th edition
PCLS: Patient PTSD checklist scale
PDEQ: Peritraumatic dissociative experience questionnaire
PPE-15: Picker patient experience
PTSD: Post-traumatic stress disorder
